# Microbiome-gut-brain axis contributes to patients and Bama miniature pigs with acute large ischemic stroke

**DOI:** 10.3389/fnins.2024.1378844

**Published:** 2024-07-12

**Authors:** Dazhi Deng, Hehua Lei, Zheng Cao, Cui Zhang, Ruichen Du, Xin Gao, Junjie Wei, Yibo Lu, Xiangzhen Zhou, Limin Zhang

**Affiliations:** ^1^Wuhan National Laboratory for Optoelectronics, Huazhong University of Science and Technology, Wuhan, China; ^2^Department of Emergency, The People's Hospital of Guangxi Zhuang Autonomous Region and Guangxi Academy of Medical Sciences, Nanning, China; ^3^State Key Laboratory of Magnetic Resonance Spectroscopy and Imaging, National Centre for Magnetic Resonance in Wuhan, Innovation Academy for Precision Measurement Science and Technology, CAS, Wuhan, China; ^4^University of Chinese Academy of Sciences, Beijing, China; ^5^Department of Neurology, The People's Hospital of Guangxi Zhuang Autonomous Region and Guangxi Academy of Medical Sciences, Nanning, China; ^6^Department of Radiology, Nanning Fourth People's Hospital and Guangxi AIDS Clinical Treatment Center, Nanning, China; ^7^Department of Pathology, The People's Hospital of Guangxi Zhuang Autonomous Region and Guangxi Academy of Medical Sciences, Nanning, China

**Keywords:** acute ischemic stroke, Bama miniature pig, gut-brain axis, plasma metabolic profiling, gut microbiota and its metabolites

## Abstract

Acute large hemispheric infarction (ALHI) is an overwhelming emergency with a great challenge of gastrointestinal dysfunction clinically. Here, we initially proposed delayed bowel movements as the clinical phenotype of strike to gut-brain axis (GBA) in ALHI patients by epidemiological analysis of 499 acute ischemic stroke (AIS) patients. ^1^H NMR-based metabolomics revealed that AIS markedly altered plasma global metabolic profiling of patients compared with healthy controls. Risk factors of strike on GBA were the National Institutes of Health Stroke Scale (NIHSS) score ≥ 5 and stroke onset time ≤ 24 h. As a result, first defecating time after admission to the hospital ≥2 days could be considered as a potential risk factor for strike on GBA. Subsequently, the ALHI Bama miniature (BM) pig model with acute symptomatic seizure was successfully established by ligation of the left ascending pharyngeal artery combined with local air injection. Clinical phenotypes of brain necrosis such as hemiplegia were examined with brain diffusion-weighted imaging (DWI) and pathological diagnosis. In addition to global brain injury and inflammation, we also found that ALHI induced marked alterations of intestinal barrier integrity, the gut microbial community, and microbiota-derived metabolites including serotonin and neurotransmitters in both plasma and multiple brain tissues of BM pigs. These findings revealed that microbiota-gut-brain axis highly contributed to the occurrence and development of ALHI.

## 1 Introduction

Stroke is a leading cause of adult disability and mortality worldwide, especially in people aged 50 years and older (WHO, 2020; Ding et al., 2022). Epidemiological studies have shown that approximately 80% of stroke patients are diagnosed with ischemic stroke (Boursin et al., 2018; Barthels and Das, 2020; Wang et al., 2022), and anterior circulation ischemic infarctions (ACIs) account for 80% of ischemic strokes cases (Sparaco et al., 2019). Acute large hemispheric infarction (ALHI) caused by the interruption of blood supply in the middle cerebral artery is a severe form of ischemic stroke and accounts for 2–8% of acute ischemic stroke (AIS) (Zha et al., 2015). More than 50% of AHLI patients experience the development of malignant cerebral edema (MCE) accompanied by blood-brain barrier (BBB) breakdown and vasogenic edema formation, which lead to mortality of up to 80% (Liebeskind et al., 2019; Lehrieder et al., 2021). Meanwhile, invasive procedures including endobronchial electrocautery (He et al., 2021), central venous catheterization (Kugiyama et al., 2018), artificial pneumothorax (Gou et al., 2019), and transbronchial needle aspiration (Van Den Plas et al., 2020) that have been widely used in clinical management for patients can induce cerebral arterial air embolism. Both kinds of strokes induce neuronal cell death, systemic inflammation, and brain regional injury and subsequent metabolic disorders. However, the pathophysiologic pathways relating to outcomes of them are still largely unknown and worth conducting further research.

One typical consequence of ALHI is primary and secondary brain injury caused by focal and global brain inflammation. In ischemic stroke, the steep termination of blood supply in a vascular territory of the brain leads to the death of neural cells yielding an ischemic core and releasing damage-associated molecular patterns (DAMPs) such as adenosine and heat shock proteins, which trigger focal brain inflammation and immune response in the injured brain region (Schuhmann et al., 2021). Subsequently, a series of events including BBB damage, oxidative stress, and mitochondrial disruption result in secondary brain injury and global brain inflammation (Shi et al., 2019). Following an acute brain injury over stroke, peripheral leucocytes infiltrate the injured brain and release proinflammatory cytokines, thus further aggravating the BBB disruption and brain injury (Huang et al., 2022). The activated microglia by cytokines and chemokines distribute in the chronic stage of stroke forming global brain inflammation (Fifield and Vanderluit, 2020). Supportive evidence of global brain inflammation after stroke could also be found in the extensive distribution of a large number of cytokines (IL-1β, IL-6, and TNF-α) in the contralateral hemisphere of animal AIS models (Zaremba and Losy, 2001; Chen et al., 2019; Endres et al., 2022). Furthermore, previous studies also showed that global brain inflammation gradually induces global vascular inflammation in both intracerebral and subarachnoid hemorrhage mice models (Singh et al., 2018; Schuhmann et al., 2023).

In addition to brain injury and global brain inflammation, stroke results in systemic alterations including cardiovascular and gastrointestinal systems. Increasing evidence suggests that stroke can cause disruption of the gut microbiota homeostasis and intestinal epithelial barrier integrity, and vice versa (Agirman et al., 2021). The gut and its microbiota could also increase the risk of cerebrovascular events highly contributing to the onset of stroke (Agirman et al., 2021). Patients with ALHI, in particular, face a high risk of gastrointestinal dysfunction with a potential impact on the microbiome-gut-brain axis (Coggrave and Norton, 2013; Arunachala Murthy et al., 2022). In clinical stroke, previous studies with relatively limited sample size identified overall 62 upregulated (e.g., *Enterobacteriaceae*, *Streptococcus*, *Lactobacillus,* and *Escherichia*) and 29 downregulated microbial taxa (e.g., *Eubacterium* and *Roseburia*) in the fecal microbial community (Xu et al., 2021; Peh et al., 2022). In experimental stroke, specific gut microbiome composition and their metabolites such as short-chain fatty acids closely contribute to the severity of stroke. Although clinical stroke is limited due to ethical restrictions that prevent invasive sampling of AIS patients (Coggrave and Norton, 2013; Xu et al., 2021), these experimental data suggested that modulation of an interplay between the gut and brain could be a novel therapeutic strategy for stroke prevention.

In this study, we first conducted an epidemiological analysis of 499 AIS patients coupled with clinical magnetic resonance imaging (MRI) and computer tomography (CT) examinations. A phenomenon of delayed bowel movements was observed in LHI patients, which may be induced by a strike to the GBA. ^1^H NMR-based metabolomics was also employed to reveal global metabolic profiling in the plasma of AIS patients. Subsequently, a Bama miniature (BM) pig model with ALHI was successfully established and employed to investigate the alterations of intestinal barrier integrity, the gut microbial community, and microbiota-derived metabolites. These findings highlight that microbiome-gut-brain axis contributes to both clinical AIS patients and ALHI BM pigs.

## 2 Materials and methods

### 2.1 Epidemiology of AIS patients in comprehensive stroke center

This clinical and randomized study was reviewed and approved by the Ethics Committee of the People’s Hospital of Guangxi Zhuang Autonomous Region in China (NO: KY-KJT-2019-34). Clinical data of AIS patients including baseline demographic characteristics and magnetic resonance imaging (MRI) were performed in the emergency department of a comprehensive stroke center from 1 February 2022 to 31 March 2023. Epidemiology of AIS patients including the time of first medical contact after symptom onset, the National Institutes of Health stroke scale (NIHSS) score, first defecating time after admission to the hospital, diagnosis, and prognosis were also conducted.

### 2.2 Statistical analysis of AIS patients’ epidemiologic data

Statistical analysis was carried out with SPSS 23.0 (Chicago, IL, United States). Enumeration data and normal distribution data were presented as percentages and mean ± standard deviation, respectively, and skewed distribution data were expressed as median (interquartile range). Baseline demographic characteristics of AIS patients were analyzed with descriptive statistics. First defecating time after admission to the hospital ≥2 days were considered as a potential risk factor for strike on GBA (Xu et al., 2021), which were identified via Spearman’s correlation matrix and binary logistic regression analysis. Group grade/assignment criteria for binary logistic regression analysis are shown in Supplementary Table S1. The dependent variable was whether the strike on brain-gut axis happened. NIHSS score = 0–1 was set as control group grade, and other NIHSS score grade, strike onset time, and age were provided in the equation. A *p*-value of < 0.05 was considered to indicate significant differences statistically. The relationship between strike on brain-gut axis and severe adverse treatment outcomes to different NIHSS score AIS patients was analyzed through the Kruskal–Wallis test, while the NHISS score grade was conformed as a risk factor.

### 2.3 Regents

Laboratory supplies including a 14G single-lumen single-use sterile central venous catheter kit (Medical Device Registration Certificate Number/Product Technical Requirements Number: State Mechanical Note Quarantine 20173664588) were purchased from Shenzhen Yixinda Medical New Technology Co. Experimental drugs include midazolam injection 5 mg/mL (Jiangsu Enhua Pharmaceutical Co., Ltd., National Drug Quarantine H19990027), 2% lidocaine hydrochloride injection 20 mg/mL (Hubei Tiansheng Pharmaceutical Co., Ltd., National Drug Quarantine H42021839), propofol medium/long-chain fatty lactic acid injection 10 mg/mL (Beijing Fresenius Kabi Pharmaceutical Co., Ltd., State Drug License HJ20150655), and sufentanil injection 50 μg/mL (Yichang Renfu Pharmaceutical Co., Ltd., State Drug License H20054171). DEHP was purchased from J&K Scientific Ltd. (Beijing, China). Fatty acids, phospholipids, and choline metabolites were obtained as internal references from Sigma-Aldrich Chemical Co. Ltd. (St Louis, MO). Chemical reagents at analytical grade including sodium chloride, K_2_CO_3_, K_2_HPO_4_, and NaH_2_PO_4_ were purchased from Sinopharm Chemical Co. Ltd. (Shanghai, China). Glutaraldehyde, sodium 3-trimethylsilyl [2,2,3,3-d4] propionate (TSP-d4), osmium tetroxide, and D_2_O (99.9% in D) were purchased from Cambridge Isotope Laboratories (Miami, FL).

### 2.4 ^1^H NMR-based plasma metabolic profiling of AIS patients and healthy controls

^1^H NMR spectra of plasma obtained from AIS patients and healthy controls were performed by a Bruker Avance III 600 MHz spectrometer on the conditions of 298 K and 600.13 MHz, equipped with an inverse detection cryogenic probe (Bruker BioSpin, Germany). Detailed information on plasma sample preparation, NMR singling acquisition, and data statistical analyses is provided in Supplementary material.

### 2.5 Animal experiment

Animal experiment was conducted in accordance with the Animal Ethics Committee of the People’s Hospital of Guangxi Zhuang Autonomous Region & Guangxi Academy of Medical Sciences (NO: KY-KJT-2019-34). A total of 12 conventional BM pigs (6 months old, 14–30 kg body weight) were purchased from the College of Animal Science and Technology, Guangxi University (NO: SCXK Gui 2018–0003, Nanning, China). Animals were randomly assigned to two groups (*n* = 6) with no significant difference in body weight comparison (18.58 ± 2.15 vs. 19.92 ± 5.46 kg).

After an intramuscular injection of 5 mg midazolam for 10–15 min and regional anesthesia with 100 mg lidocaine hydrochloride for 1–3 min, a central venous catheter (CVC) with a depth of 8–10 cm was implanted to anterior vena cava of animal through right supraclavicular fossa when the animal was fastened in the supine position with hyperextension. For general anesthesia, intravenous anesthetic induction with 20 mg propofol medium/long-chain fat emulsion injection and 2 μg sufentanil injection was performed through a CVC, followed by 20–40 mg/h propofol medium/long-chain fat emulsion intravenous injection to maintain anesthesia. Rectal temperature was 38.3–38.9°C, heart rate was 76.0–107.0 times/min, respiratory rate was 13.0–42.0 times/min, and pulse oxygen saturation (SPO_2_) was 95.0–100.0%, respectively, during the period of anesthesia while oxygen inhalation was carried out.

### 2.6 Establishment of animal LHI models

A 3- to 5-cm longitudinal incision deep to the surface of the muscular layer was made in the anterior region of the neck 3 cm to the left side of the midline at the level of the mandibular angle and the upper edge of the thyroid cartilage with an animal in the supine position. The muscular layers were separated bluntly until the left carotid sheath was exposed. Then, the left common carotid artery in the left carotid sheath was dissected cephalad until the bifurcation of the left ascending pharyngeal artery and the left external carotid artery was exposed. In the model group, the external carotid artery and the proximal common carotid artery were ligated, and the distal common carotid artery was cut with scissors in a “V” shape. The expanding tube in the CVC set was placed into the distal ascending pharyngeal artery, and 0.125–0.18 mL/kg air was injected slowly until the animal got an acute seizure of epilepsy including symptoms of convulsions and limb stiffness. Then, the expanding tube was pulled out, the distal side of the common carotid artery was ligated, and muscular layers, subcutaneous tissue, and skin were intermittently sutured, respectively. Finally, the animal was placed on an electric blanket in the lateral position because of a neurological deficit. Animals in the control group received only dissection of the common carotid artery and its branches.

### 2.7 Neurological deficit score for pigs

The neurological deficit score of animals was evaluated by two medical doctors when the operation was finished and re-evaluated in 3, 6, and 9 h after symptom onset with a table of neurological deficit score for pigs as previously described (Wenzel et al., 2000). The manifestations of neurological deficit in pigs were clonus and rigidity of limbs, bilateral nystagmus initially, added with paralysis of the left hindlimb promptly and eventually. Therefore, pigs in the model group got 70 points because it could not stand (20 points) or walk (30 points) and got sluggish resists (20 points) over 9 h since symptom onset.

### 2.8 Brain magnetic resonance imaging

Magnetic resonance imaging (MRI) for pig brains including diffusion-weighted imaging (DWI) and magnetic resonance angiography (MRA) were acquired with MAGNETOM ESSENZA 1.5 T magnetic resonance scanner (SIEMENS, Germany) 4 h after symptom onset or sham operation under general anesthesia. Analysis of MRI was finished with Syngo via imaging software by two Radiologists. In brief, the heads of pigs were put into eight-channel orthogonal head coil and scanned with the following parameters in the prone position. DWI transverse sequence was selected as a single excitation plane echo, and two diffusion gradient factor b values (0 s/mm, 1,000 s/mm) were selected to reconstruct the apparent diffusion coefficient (ADC) map: repetition time (TR) 4,100 ms, echo time (TE) 129 ms, scan field 230 mm × 230 mm, resolution 130 × 130, slice thickness 6 mm, slice spacing 1.2 mm, total number of slices 20, and acquisition time 55 s. The MRA scanning method was three-dimensional time of flight (3D-TOF): TR 26 ms, TE 6.8 ms, flip Angle 20°, scan field 230 mm × 200 mm, resolution 300 × 300, slice thickness 1 mm, slice spacing −23.33 mm, total number of slices 70, and acquisition time 3 min 23 s. After symptom onset or sham operation for 9 h, pig blood was collected through CVC, and plasma samples were stored at −80°C for later analyses after centrifugation at 0°C. All BM pigs were sacrificed under general anesthesia, and ileocecal contents were collected and stored at −80°C for the following experiments. Partial brain and ileocecum tissues were fixed with 10% formalin solution for histopathological examinations or immunofluorescence analysis, and the rest was stored at −80°C for later analyses.

### 2.9 Histopathological assessments

The partial left parietal lobe of brain samples was formalin (10%)-fixed and embedded in paraffin. Sectioned tissue (5 μm) was stained with hematoxylin and eosin and then evaluated by two professional pathologists with a microscope (Leica DM4000 B LED, Germany).

### 2.10 Quantitative real-time PCR analysis

The procedures of RNA extraction from the partial left parietal lobe of the brain and ileocecum tissues, cDNA synthesis, and data analyses are described in Supplementary material. The data were analyzed using the ΔΔCT method. Primer sequences of genes (*IL-1β*, *TNF-α*, *ZO-1*, and *E-cadherin*) are shown in Supplementary Table S2.

### 2.11 Measurement of plasma lipopolysaccharides

Pig plasma lipopolysaccharide (LPS) concentration was measured with an LPS ELISA Kit (Nanjing Jiancheng Bioengineering Research Institute Co., LTD., China) according to the product description.

### 2.12 Confocal immunofluorescent analysis

Partial ileocecum tissue was formalin (10%)-fixed and embedded in paraffin. Sectioned tissues (5 μm) were incubated with primary antibodies, rabbit anti-ZO-1 catalog # 21773-1Ap (1:1,500, Proteintech), mouse anti-E-cadherin Catalog #14472 (1,200, CST) antibodies in fresh blocking solution and incubated with ileocecum slides for overnight at 4°C. After washing with PBS, ileocecum slides were incubated with secondary antibodies (goat anti-rabbit, 1:500, goat anti-mouse, 1:1,000, Thermo Fisher) conjugated with fluorescent groups. Ileocecum slides were mounted to glass slides and photographed by Zeiss Pascal confocal microscope (Carl Zeiss, United States). Zeiss LSM image browser software (Carl Zeiss, United States) and Image J were used for the analysis of images.

### 2.13 Gut microbiota analysis by 16S rRNA gene sequencing

The total DNA of gut bacteria was extracted from ileocecal contents (~50 mg), and the amplicon sequence library of the 16S rRNA gene was constructed according to the 16S metagenomic sequencing library preparation protocol (Illumina, United States). The V3–V4 region of the 16S rRNA gene was amplified using a Kapa HiFi HotStart PCR Kit (KAPA Biosystems, United States). Double-index barcodes were added to amplicon targets by exponential PCR using the Nextera^®^ Index kit (United States). Amplicons were purified with AMPure XP beads and quantified using the KAPA Library Quantification Kit (KAPA Biological Systems, United States). Equimolar amounts of purified amplicons were pooled, and paired-end sequencing (2 × 300 bp) was performed using the illumination platform of Shanghai Mayo Bio Biotechnology Co., LTD. We used QIIME (version 1.9.1) to demultiplex the sequences, quality filter (Phred quality score ≥ 20), and delete singleton sequences and then used the SILVA_132 database to construct OTU tables based on the open reference UCLUST method. The UCLUST and SILVA_132 databases were used for the taxonomic assignment of these OTUs. Most statistical analyses and data manipulation were performed using the ggplot2 software package. Each sample was refined to 35,380 reads before diversity analysis. α-diversity (observed OTUs) was calculated using the R software using both vegans and fossils. Differences in community composition between samples (β-diversity) were represented by Bray–Curtis differences calculated from OTU abundance, and principal coordinate analysis (PCoA) was used to show and compare patterns of microbial communities. The OTUs proposed by the heatmap were analyzed by hierarchical clustering using the heatmap package and R software.

### 2.14 Quantitative analysis of plasma and brain tryptophan metabolites

The targeted quantitative measurement of tryptophan was finished on an Agilent 6,460 triple-quadrupole mass spectrometer (UHPLC-QQQMS) coupled with an UHPLC column (Agilent Zorbax Eclipse XDB-C18 Rapid Resolution HD, 2.1 m × 100 mm, 1.8 μm). Quantification of tryptophan metabolites was conducted on a UHPLC–MS/MS system equipped with a UHPLC column (ACQUITY UHPLC BEH Amide column, 2.1 m × 100 mm, 1.7 μm). Procedures for sample preparation and quantification of tryptophan metabolites measurements were described previously and in the Supplementary material.

### 2.15 Quantitative analysis of plasma and brain neurotransmitter metabolites

Neurotransmitter metabolites were extracted from plasma and brain tissues using methanol with deuterated internal standards. Qualitative and quantitative analyses were performed on an Agilent 1290 ultrahigh-performance liquid chromatography system coupled with an Agilent 6460 triple-quadrupole mass spectrometer (UHPLC-QQQ-MS, Agilent Technologies, Inc.). The sample (1 μL) was individually injected on an ACQUITY UPLC BEH Amide column (1.7 μm, 2.1 mm × 100 mm, Waters Corporation). The column temperature was 40°*C. mobile* phase A contained 10 mM ammonium formate and 0.1% formic acid in 50% acetonitrile and 50% water. Mobile phase B contained 10 mM ammonium formate and 0.1% formic acid in 90% acetonitrile and 10% water. The gradient used for the analysis was from 100% B to 100% A over 5 min and held in 100% A for 2 min. Mass spectra were acquired in positive multiple reaction monitoring (MRM) mode. Quantification of neurotransmitter metabolites was performed using calibration curves and internal standards.

### 2.16 Statistical analysis for animal experimental data

Statistical data analyses were carried out in GraphPad Prism (version 8.0) and presented as mean ± SD. One-way ANOVA followed by Tukey’s *post-hoc* test was performed for comparisons between the two groups. For all experiments, a *p*-value of < 0.05 was set as the statistical significance.

## 3 Results

### 3.1 Epidemiology of AIS patients

The baseline demographic characteristics of patients with AIS are shown in Table 1. A total of 499 patients with AIS included 411 (82.4%) acute cerebral infarction (ACI) and 352 male patients (70.5%). The median (IQR) of AIS patients age, stroke onset time, and NIHSS score were 65 (58–73) years, 24 (11–38) h, and 4 (2–6), respectively. Among the 499 AIS patients assessed, 341 (68.3%) had comorbid hypertension and/or diabetes mellitus, and 26 (5.2%) had atrial fibrillation. The median (IQR) NIHSS score was 4 (2–6), with a range of 0–33 (mean ± SD: 4.99 ± 4.21). Here, AIS patients were grouped according to NIHSS score as previously described (Chalos et al., 2020); 187 cases (37.5%) were classified as moderate stroke due to NIHSS scores ranging from 5 to 15, and 20 (4%) were classified as moderately severe stroke due to NIHSS scores greater than 15. The median of first defecating time after admission was 2 (1–3) days. Of the patients, 182 cases (36.5%) exhibited symptoms of GBA dysfunction, defined as initial defecation occurring after ≥2 days of hospitalization. The Spearman correlation coefficient matrix between strike of GBA and its risk factors related to stroke (Table 2) indicated that stroke onset time had a negative correlation with age and NIHSS score (−0.133, *p* < 0.01, and −0.114, *p* < 0.05, respectively). However, first defecating time and stroke onset time had a negative correlation with stroke onset time (−0.105, *p* < 0.05) and a positive correlation with NIHSS score (0.281, *p* < 0.01), respectively (Table 2). The binary logistic regression analysis results showed that there was a statistically significant increase in the incidence of cerebral-intestinal axis damage among stroke patients. Specifically, the mild stroke group showed a 2.067 times higher incidence (*p* < 0.05), the moderate stroke group showed a 3.522 times higher incidence (*p* = 0.01), and the moderately severe stroke group showed a 23.315 times higher incidence (*p* = 0.01) than the corresponding control group. The occurrence of involvement in the cerebral-gut axis was 2.076 times higher within 24 h of a stroke compared to more than 24 h after (*p* = 0.01) (Table 3). Kruskal–Wallis test results further demonstrate that severe adverse treatment outcomes display significant differences in NIHSS score grades: *H* = 87.375, *p* = 0.001 (Table 4).

**Table 1:** Baseline demographic characteristics of AIS patients (*N* = 499).

Variables	Cases
ACI, n/N (%)	411 (82.4%)
Men, n/N (%)	352 (70.5%)
Age in years, median (IQR)	65 (58–73)
**Stroke onset time in hours, median (IQR)**	24 (11–38)
**With guidelines recommended treatment, n/N (%)**	48 (9.6%)
≤3–6H, Intravenous thrombolysis, n/N (%)	42 (8.4%)
<24H, endovascular treatment, n/N (%)	4 (0.8%)
=3H, intravenous thrombolysis and sequential endovascular treatment, n/N (%)	2 (0.4%)
**Without intravenous thrombolysis or endovascular treatment, n/N (%)**	451 (90.4%)
<3H, n/N (%)	7 (1.4%)
3–6H, n/N (%)	23 (4.6%)
≤24H, n/N (%)	273 (54.7%)
>24 h	148 (29.7%)
**Comorbidity**	
Hypertension and/or diabetes mellitus	341 (68.3%)
Atrial fibrillation, n/N (%)	26 (5.2%)
**NIHSS score, median (IQR)**	4 (2–6)
0–1, normal group	74 (14.8%)
1–4, mild stroke group	218 (43.7%)
5–15, moderate stroke group	187 (37.5%)
>15, Moderately severe group	20 (4%)
First defecating time in day (IQR)	2 (1–3)
Strike on brain-gut axis *, n/N (%)	182 (36.5%)

IQR, interquartile range presented as the 25th and 75th percentile; * first defecating time after admission to the hospital ≥ 2 days were considered as striking on brain-gut axis.

**Table 2:** Spearman’s correlation matrix results about the risk factor of stroke.

Item	Age	Stroke onset time	NIHSS score	First defecating time
Age	1			
Stroke onset time	−0.133**	1		
NIHSS score	0.085	−0.114*	1	
First defecating time	0.081	−0.105*	0.281**	1

*p < 0.05; **p < 0.01.

**Table 3:** Binary logistic regression analysis results with risk factors of stroke.

Variables	*B*	Standard error	Wald *X*^2^	*p*	OR (95%CI)
**NIHSS score**			28.596	0.001	
0–1, control				–	1
2–4, mild	0.726	0.334	4.724	0.030	2.067 (1.074–3.978)
5–15, moderate	1.259	0.336	14.082	0.001	3.522 (1.825–6.799)
≥15, moderately severe	3.149	0.700	20.237	0.001	23.315 (5.913–91.939)
Strike onset time (≤24H vs. >24H)	0.730	0.228	10.307	0.001	2.076 (1.329–3.243)
Age in years	0.013	0.009	1.945	0.163	1.013 (0.995–1.031)

In the equation, whether the strike on brain-gut axis happened was the dependent variable (Yes = 1, No = 0).

**Table 4:** Severe adverse treatment outcomes with different NIHSS scores.

NIHSS score	Treatment outcome		Mean rank	*H*	*p*
	Death/coma/hemiplegia	Others			
0–1, normal	1 (1.35%)	73 (98.65%)	261.63	87.375	0.001
1–4, mild	1 (0.46%)	217 (99.54%)	263.86		
5–15, moderately severe	18 (9.63%)	169 (90.37%)	240.98		
>15, moderately severe	10 (50%)	10 (50%)	140.25		

### 3.2 Brain imaging changes in patients and BM pigs with LHI

**Figure 1:**
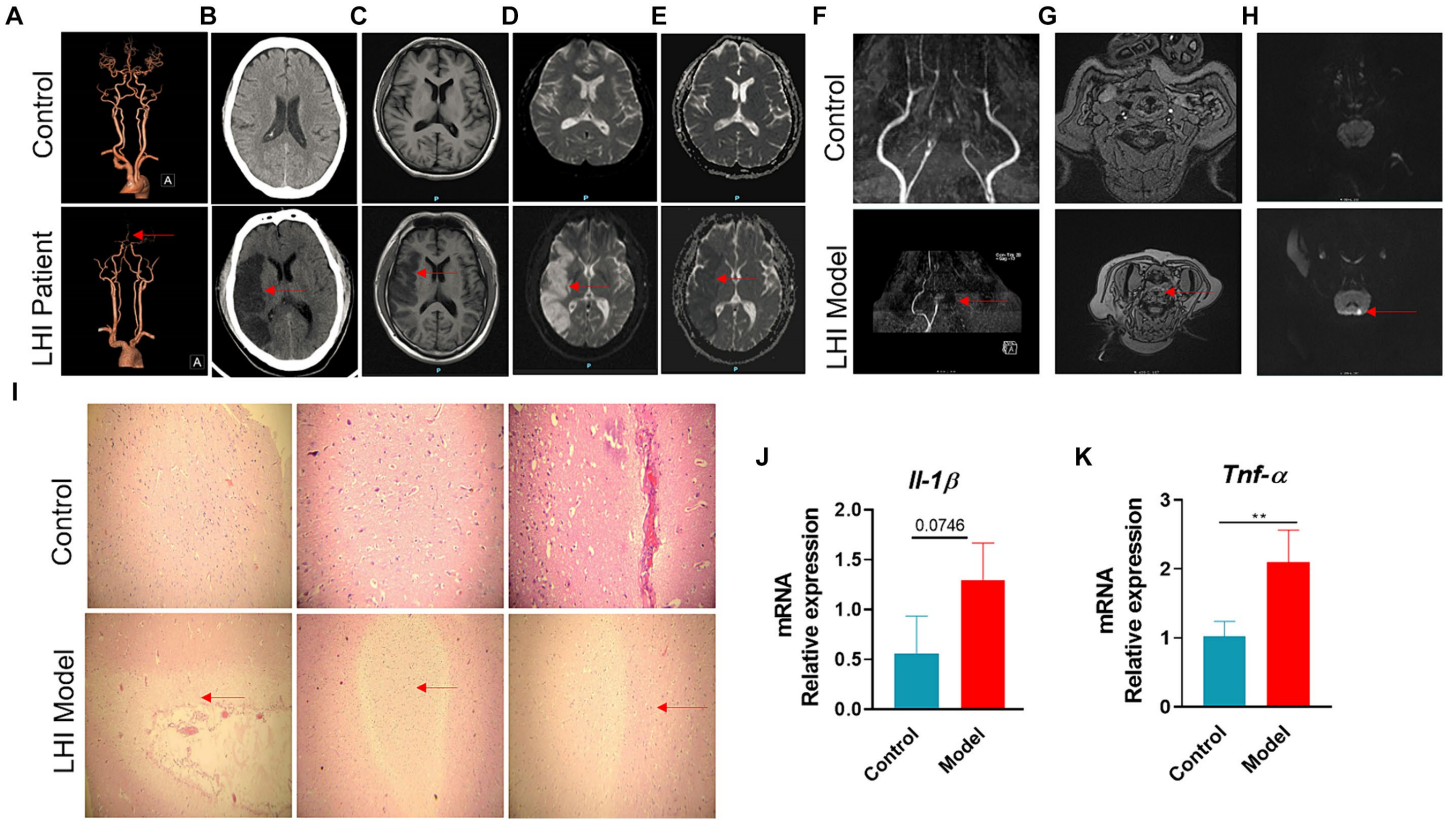
Typical vessel and brain-damaged images of AHLI patient and BM pigs. Right middle cerebral artery M2 segment occlusion of AHLI patient shown with CTA **(A)**, large fragment necrosis caused by the occlusion shown with CT **(B)**, T1WI **(C)**, DWI **(D)**, and ADC **(E)**. Left internal carotid artery occlusion of BM pigs shown with TOF-MRA in coronal section **(F)** and transverse plane **(G)**, initial large fragment necrosis caused by the occlusion shown with DWI **(H)**. **(I)** Light micrographs of brain H&E-stained sections of AHLI BM pigs, arrows represent cell edema and focal necrosis (bar = 200 μm); **(J)** brain mRNA levels of inflammatory factors including *Il-1β* and *Tnf-α* **(K)**. Data are shown as mean ± SD; *n* = 6 per group. *p < 0.05, **p < 0.01, and ***p < 0.001, by two-tailed Student’s *t*-test or Welch’s *t*-test.

To monitor the onset of symptoms of LHI, a 50-year-old patient with a significant hemispheric infarction who did not receive adequate treatment for personal reasons was selected for continuous examination. The occlusion of the middle cerebral artery for 4 days resulted in typical vascular and cerebral damage (Figure 1). The patient was hypertensive and presented with symptoms 10 h prior to admission. During the initial visit, the NIHSS score was 6. The patient had his first bowel movement on the fourth day of admission. The coronal section of the head and neck spiral CT angiography revealed that the M2 segment, along with its distal portion in the right middle cerebral artery, could not be observed, indicating occlusion (Figure 1A). Large flakiness and patchiness with abnormal signals were observed in the supply area of the right middle cerebral artery in the coronal section of the plain CT scan of the brain, with clearly defined borders (Figure 1B). MRI provides T1-weighted images of coronal sections (Figure 1C), diffusion-weighted imaging (DWI) (Figure 1D), and apparent diffusion coefficient (ADC) maps (Figure 1E), all of which indicate identical regions of necrosis.

Brain MRI changes LHI were also performed in BM pigs at LHI for 4 h after the onset of symptoms (Figures 1F–H). The coronal section of the brain using TOF (time-of-flight) MRA showed that the left internal carotid artery and its distal branches could not be observed, while the contralateral internal carotid artery in the model group had two intracranial branches. The control group had bilateral internal carotid arteries with two or three intracranial branches (Figure 1F). The same occluded intracranial branches of the left internal carotid artery are seen in the transverse plane with TOF-MRA (Figure 1G). DWI showed an area of necrosis in the left hemisphere (Figure 1H). Furthermore, H&E staining of the brain sections showed mild dilatation of blood vessels in the LHI model group, surrounded by a large number of edematous, degenerated, or even liquefied necrotic cells (Figure 1I). Consequently, patients and BM pigs with LHI experienced a marked increase in brain inflammation, manifested by a significant increase in the mRNA levels of inflammatory factors *Il-1β* and *Tnf-α* in the brain (Figures 1J,K).

### 3.3 Plasma NMR-based metabolome alteration of AIS patients

**Figure 2:**
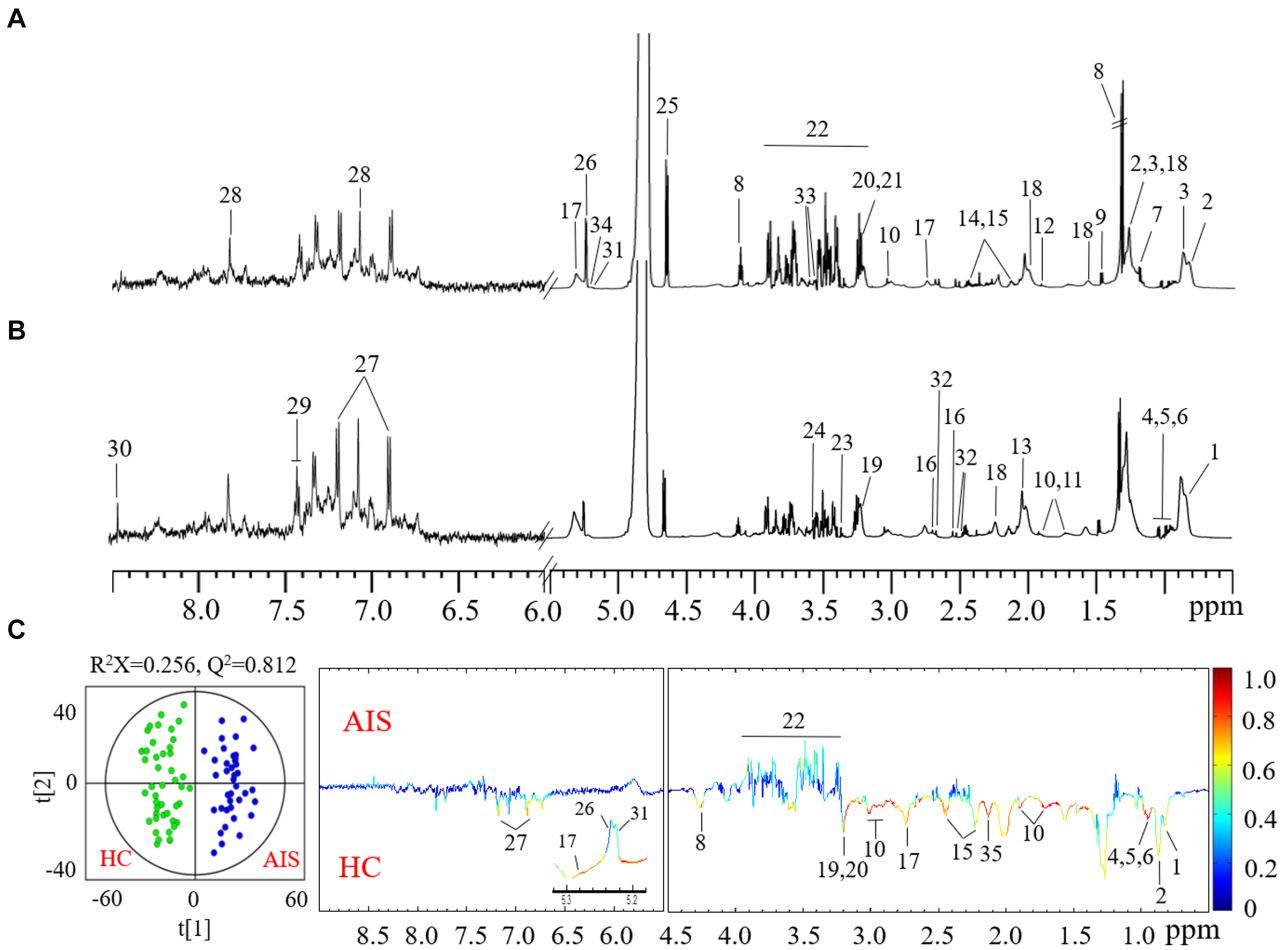
Typical ^1^H CPMG NMR spectra of plasma from healthy controls **(A)** and acute ischemic stroke (AIS) patients **(B)**. Metabolite keys: 1. high-density lipoprotein (HDL); 2. low-density lipoprotein (LDL); 3. very low-density lipoprotein (VLDL); 4. isoleucine; 5. leucine; 6. valine; 7. D-3-hydroxybutyrate (3-HB); 8. lactate; 9. alanine; 10. lysine; 11. arginine; 12. acetate; 13. N-acetyl-glycoproteins (NAG); 14. glutamine; 15. glutamate; 16. citrate; 17. succinimide; 18. lipids; 19. choline; 20. phosphorylcholine; 21. glycerophosphocholine; 22. scyllitol; 23. glucose and α-protons of amino acids; 24. glycine; 25. β-glucose; 26. α-glucose; 27. unsaturated fatty acids (UFA); 28. tyrosine; 29. phenylalanine; 30. histidine; 31. formate. 32. α-mannose; 33. acetylcarnitine; 34. myo-inositol; 35. triglycerides. **(C)** OPLS-DA scores (left) and loadings plots (right) for AIS patients with healthy controls (black) and AIS (red) (*p* < 0.05 from CV-ANOVA). The results were from the 6-fold cross-validated models, and colored scales were for the correlation coefficients (|r|) of variables.

To globally assess the metabolic profiling of AIS patients, we next examined the plasma metabolome alteration of AIS patients compared with healthy controls using an NMR-based metabolomics approach coupled with multivariate data statistical analysis. A total of 34 plasma metabolites were identified according to the NMR spectra (Figures 2A,B) and predominant by lipid, amino acid, glucose, and some organic carboxylic acids and alcohols (Supplementary Table S3). The dominantly differential metabolites were shown in the orthogonal projection to latent structure with discriminant analysis (OPLS-DA) coefficient plots (Figure 2C). Compared with the healthy control group, AIS patients exhibited significantly lower levels of low-density lipoprotein (LDL), unsaturated fatty acids, branched amino acids (leucine, isoleucine, and valine), lysine, glutamine, tyrosine, and choline metabolites together with higher levels of glucose in plasma (Figure 2C).

### 3.4 Gastrointestinal outcomes of BM pigs with LHI

**Figure 3:**
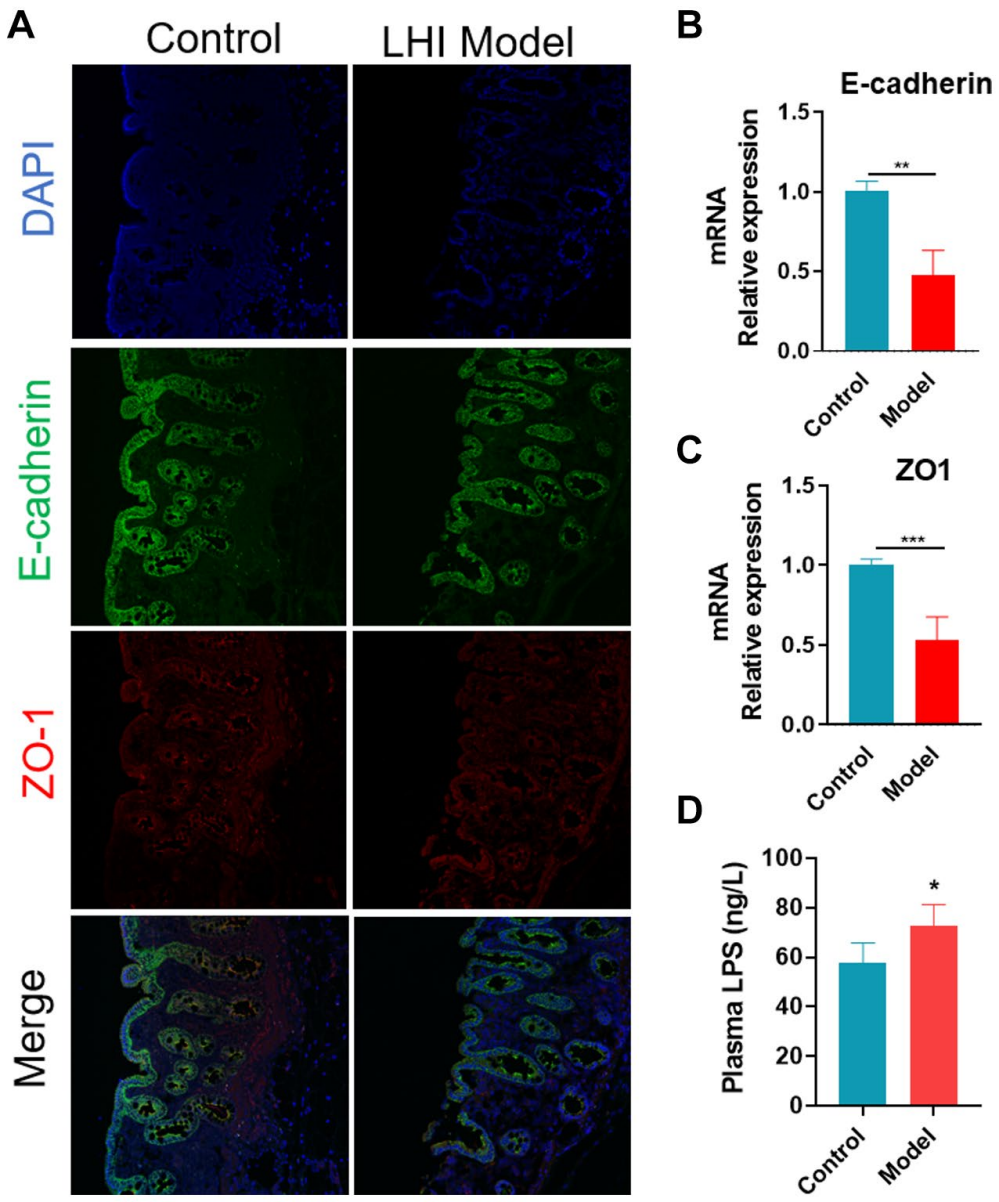
Disruption of intestine barrier integrity of BM pigs with AHLI. **(A)** Immunofluorescence imaging of mucosa thickness (DAPI, blue; E-cadherin, green; ZO-1, red). mRNA levels of E-cadherin **(B)** and ZO-1 **(C)**. **(D)** LPS concentration in plasma. *p < 0.05, **p < 0.01, and ***p < 0.001, by two-tailed Student’s *t*-test or Welch’s *t*-test.

Immunofluorescence analysis further showed that the fluorescence intensity of tight junctions (TJ) proteins (ZO-1 and E-cadherin) was attenuated in the intestine of the LHI model group (Figure 3A). Consistently, the mRNA levels of connexin (ZO-1 and E-cadherin) in the intestine were significantly reduced in the LHI model group in comparison with controls (Figures 3B,C). These results suggested that LHI induced markedly intestinal barrier dysfunction due to the GBA. Thus, endotoxin lipopolysaccharide (LPS), one of the microbial products, is eventually released from the gut lumen into the blood, leading to a high level of LPS in the plasma of BM pigs (Figure 3D).

**Figure 4:**
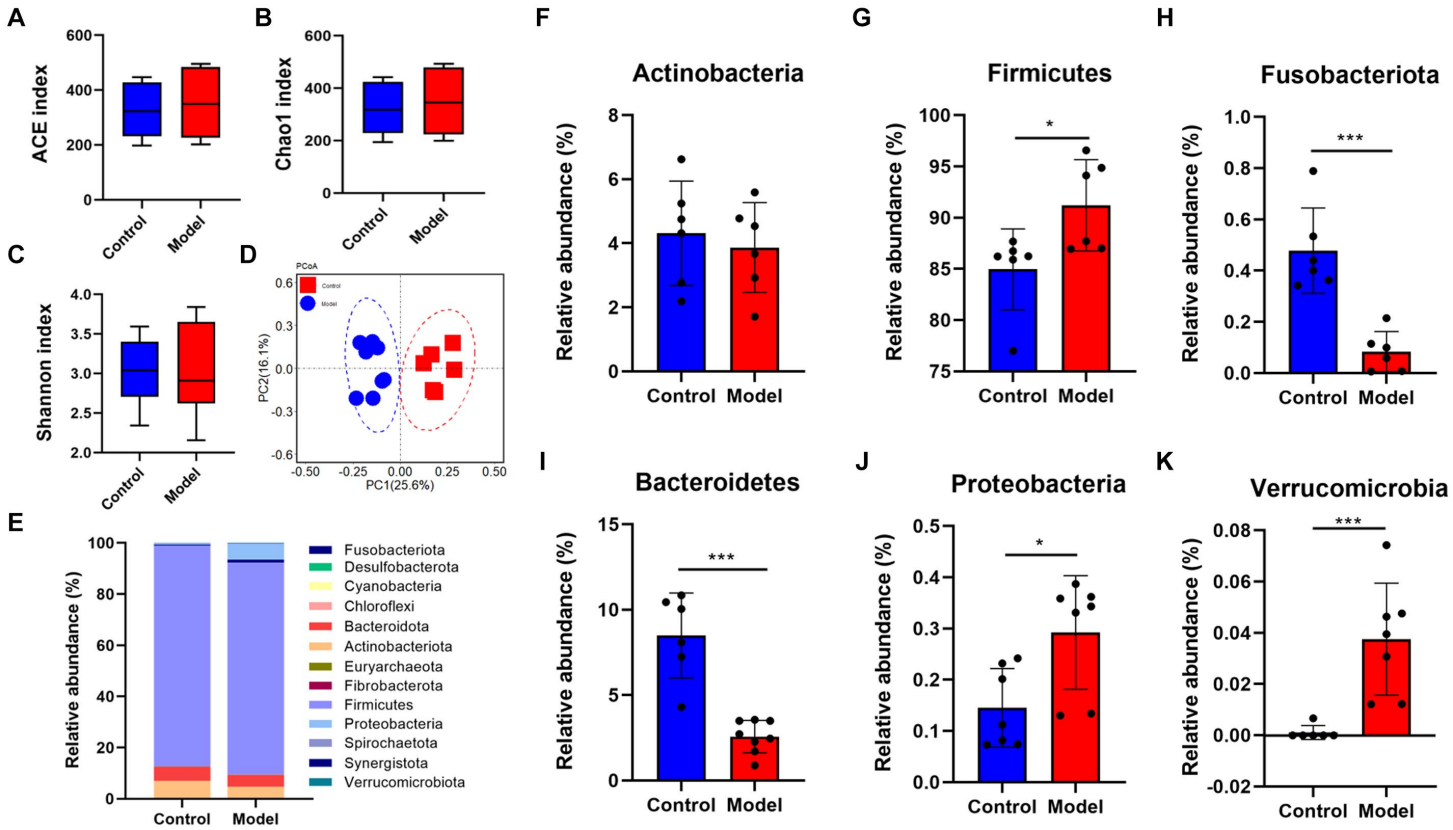
Disruption of intestine microflora profile of AHLI BM pigs at the phylum level. Microbial α-diversity **(A–D)** β-diversity and detailed contents of individual bacteria **(E–K)**. Data are shown as mean ± SD; *n* = 6 per group. *p < 0.05, **p < 0.01, and ***p < 0.001, by two-tailed Student’s *t*-test or Welch’s *t*-test.

**Figure 5:**
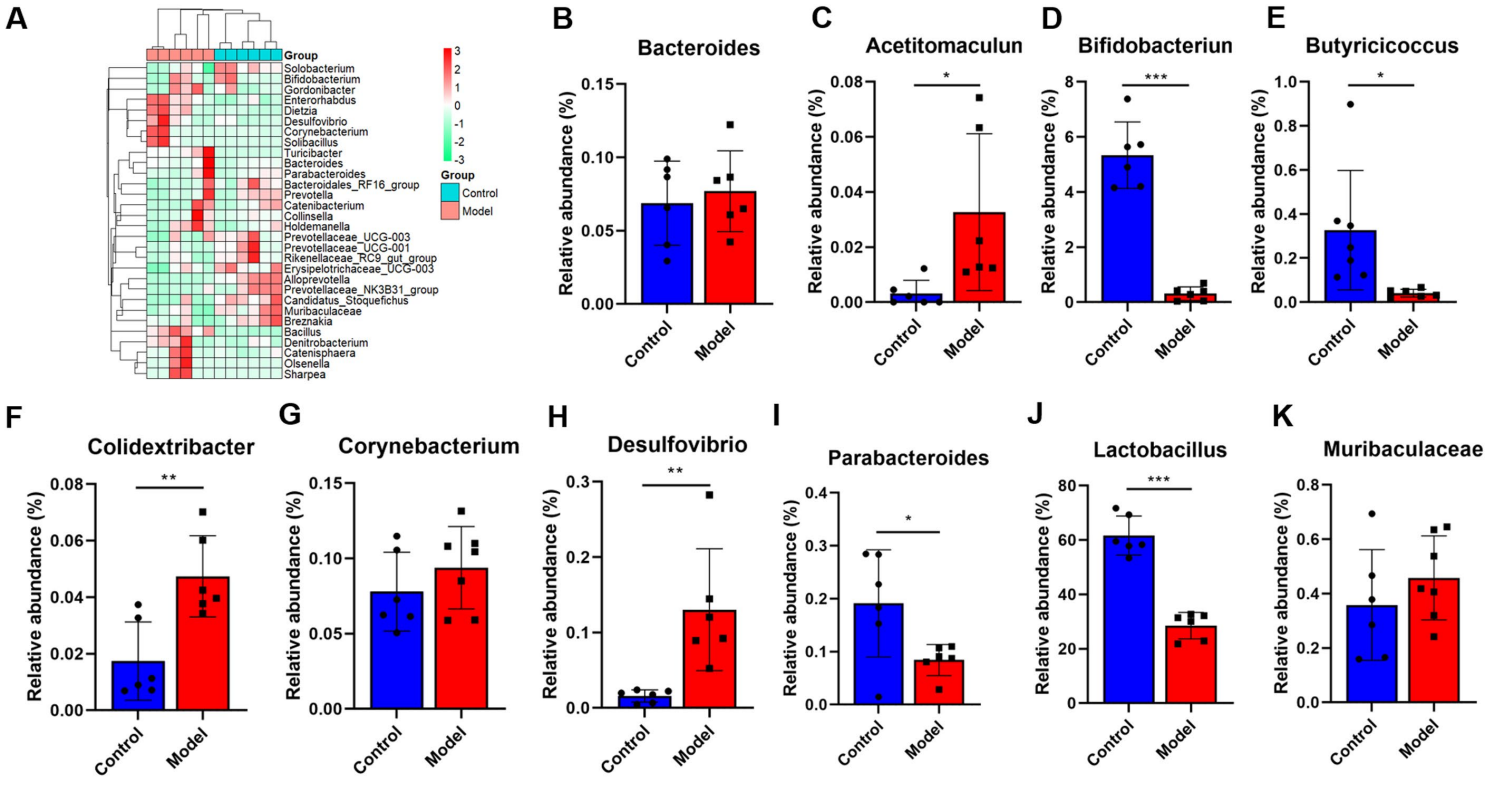
**(A–K)** The gut microflora changes of AHLI BM pigs at the genus level. Data are shown as mean ± SD; *n* = 6 per group. *p < 0.05, **p < 0.01, and ***p < 0.001, by two-tailed Student’s *t*-test or Welch’s *t*-test.

Given that patients with LHI have symptoms such as bowel dysfunction, we further analyzed the microflora profiling. 16S rRNA gene sequencing analysis showed that there was no significant change in gut microbial α-diversity, shown with no significant change in ACE index and Chao 1 (Figures 4A–C). Microbial β-diversity analysis showed that there was a clear separation between control and model groups (Figure 4D), suggesting distinct differences in the total population between them (Figure 4E). Specifically, at the phylum level, LHI models exhibited marked downregulations of the abundance of Actinobacteriota, Fusobacteriota, and Bacteroidetes and upregulation of Firmicutes, Proteobacteria, and Verrucomicrobia (Figures 4F–K). At the genus level (Figure 5A), significant upregulation in the relative abundance of *Bacteroides*, *Acetitomaculun*, *Colidextribacter*, *Corynebacterium*, *Muribaculaceae,* and *Desulfovibrio* (Figures 5B–G) together with downregulation of *Butyricicoccus*, *Parabacteroides*, *Lactobacillus,* and *Bifidobacterium* were observed in the cecal content of LHI BM pigs (Figures 5H–K).

### 3.5 Alterations in microbiota-related metabolites of BM pigs with LHI

**Figure 6:**
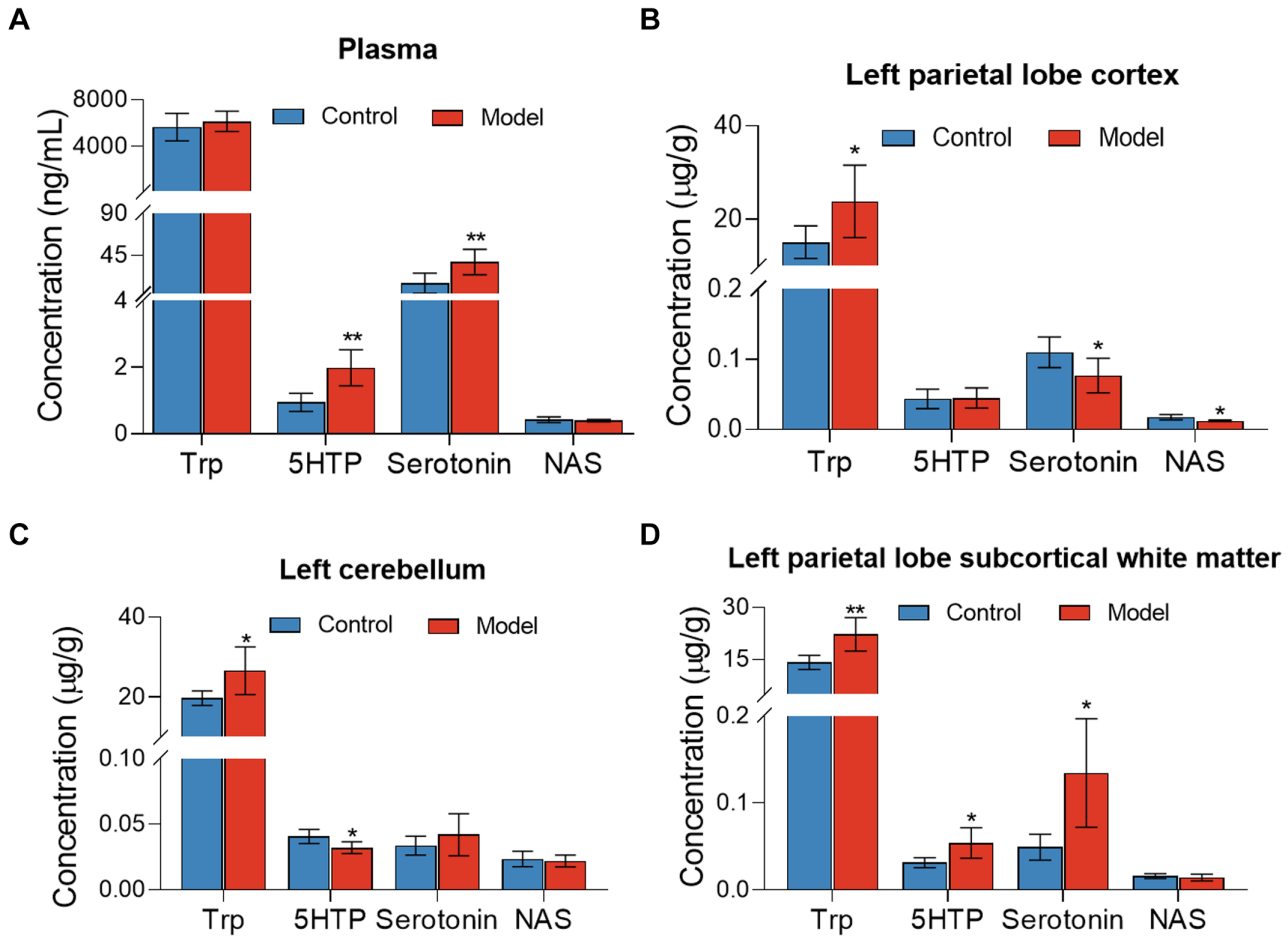
Tryptophan metabolites in plasma and multiple brain tissues of AHLI BM pigs. **(A)** Plasma; **(B)** left parietal lobe cortex; **(C)** left cerebellum; **(D)** left parietal lobe subcortical white matter. Data are shown as mean ± SD; *n* = 6 per group. *p < 0.05, **p < 0.01, and ***p < 0.001, by two-tailed Student’s *t*-test or Welch’s *t*-test.

**Figure 7:**
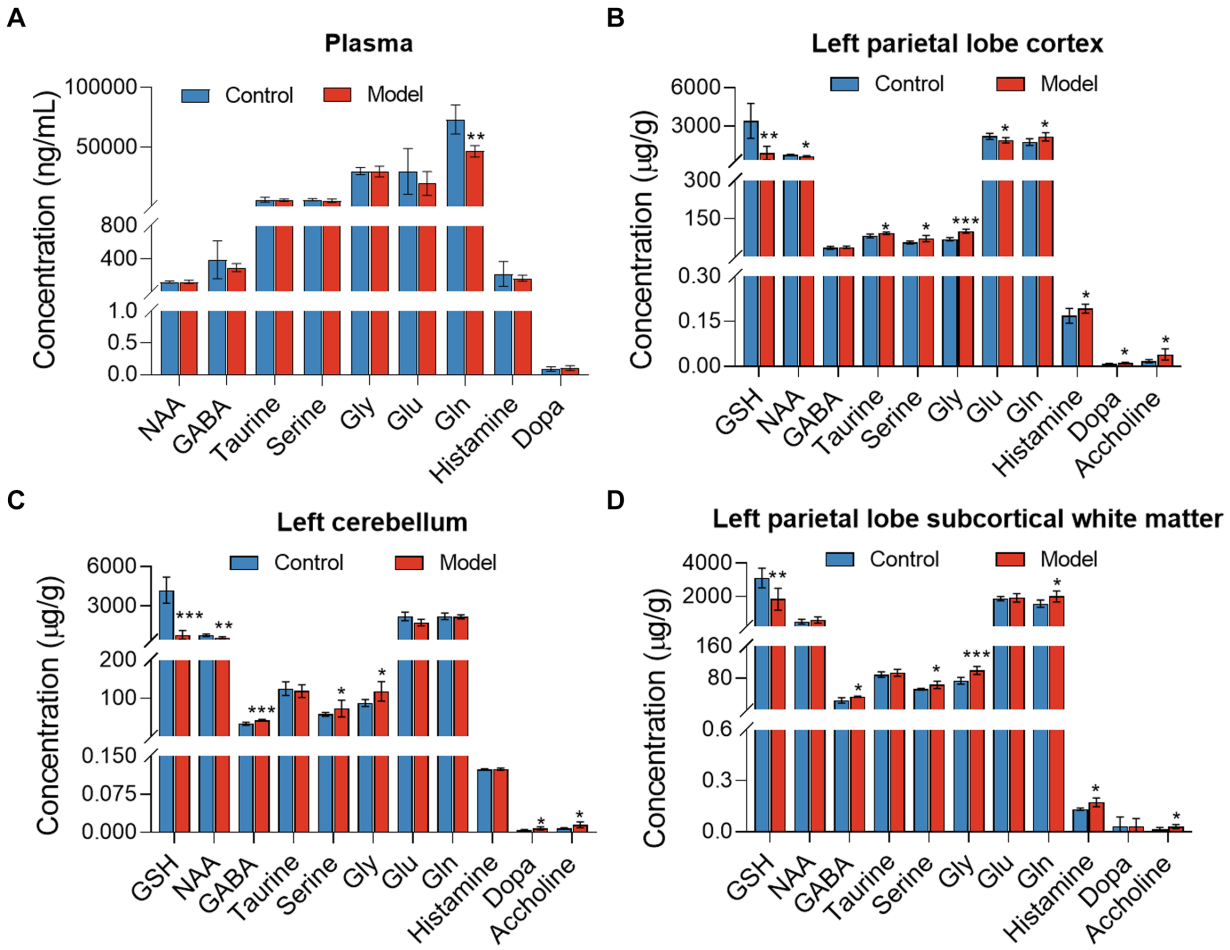
Neurotransmitter metabolism in plasma and multiple brain tissues of AHLI BM pigs. **(A)** Plasma; **(B)** left parietal lobe cortex; **(C)** left cerebellum; **(D)** left parietal lobe subcortical white matter. Data are shown as mean ± SD; *n* = 6 per group. *p < 0.05, **p < 0.01, and ***p < 0.001, by two-tailed Student’s *t*-test or Welch’s *t*-test.

**Figure 8:**
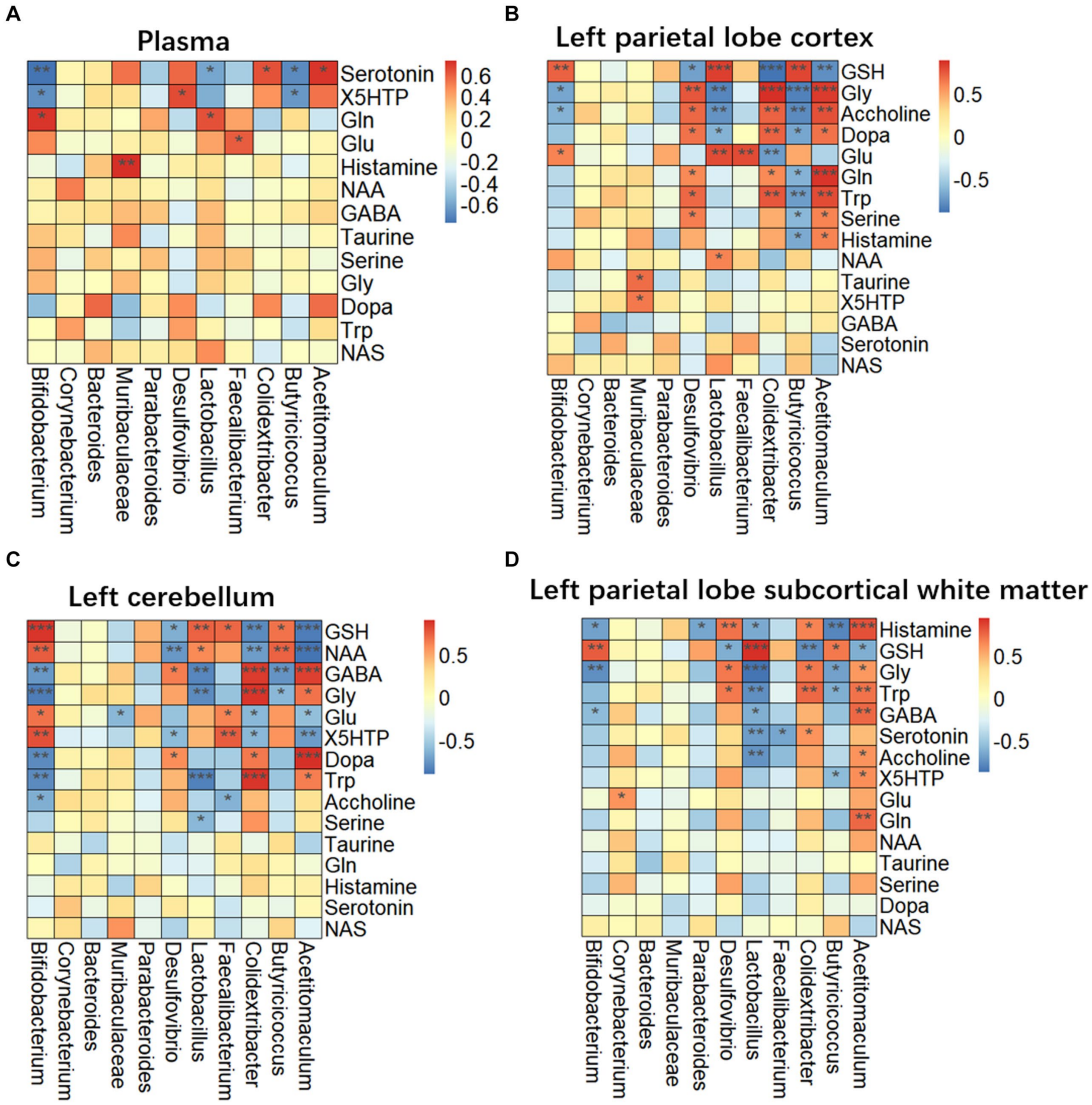
Correlation of microbiome and metabolites in plasma and multiple brain tissues of AHLI BM pigs. **(A)** Plasma; **(B)** left parietal lobe cortex; **(C)** left cerebellum; **(D)** left parietal lobe subcortical white matter. Data are shown as mean ± SD; *n* = 6 per group. *p < 0.05, **p < 0.01, and ***p < 0.001, by two-tailed Student’s *t*-test or Welch’s *t*-test.

Targeted metabolomics showed a substantial rise in plasma concentrations of 5-hydroxytryptophan (5-HTP) and serotonin in feces of the LHI model group compared with controls (Figure 6A). Furthermore, alterations in tryptophan metabolism were also observed in multiple brain components of BM pigs (Figures 6B–D). Specifically, a notable rise in tryptophan (Trp) levels alongside significant reductions in serotonin and N-acetylserotonin (NAS) was observed in the left parietal lobe cortex of BM pigs (Figure 6B). Similarly, tryptophan levels increased, while 5-HTP levels decreased in the left cerebellum (Figure 6C). In addition, the left parietal lobe subcortical white matter experienced significant increases in the levels of tryptophan, 5-HTP, and serotonin (Figure 6D). Meanwhile, the levels of some neurotransmitters metabolites also markedly changed in plasma (Figure 7A) and brain tissues (Figures 7B–D). Significant reduction in the levels of glutathione (GSH), glutamate (Glu), and glucose together with marked elevation in the levels of NAA, taurine, serine, histamine, glutamine (Gln), dopa, acetylcholine, and glycine (Gly) were observed in the left parietal lobe cortex of LHI BM pigs (Figure 7B). In the left cerebellum, LHI BM pigs exhibited significant decreases in the levels of GSH and NAA and significant increases in the levels of GABA, serine, Gly, dopa, and acetylcholine (Figure 7C). In left parietal lobe subcortical white matter, the levels of GABA, serine, Gln, histamine, acetylcholine, and Gly were significantly increased, and the level of GSH was markedly decreased (Figure 7D). Pearson’s correlation analysis further showed that marked changes of gut bacteria were more or less negatively or positively related to these Trp metabolites and neurotransmitters in multiple brain tissues of LHI BM pigs (Figures 8A–D).

## 4 Discussion

In 2021, the European Stroke Organization (ESO) has revised recommendations on intravenous thrombolysis within 9 h of symptom onset as patients with AIS (Berge et al., 2021) based on clinical determination of core/perfusion mismatch of ischemic penumbra (Albers et al., 2018; Nogueira et al., 2018). Herein, the epidemiology of 499 AIS patients revealed that 182 (36.5%) of them exhibited initial defecation occurring after ≥2 days of hospitalization, indicating symptoms of GBA dysfunction. Spearman’s correlation matrix analysis also showed that the first defecating time had a positive relationship with NIHSS score and negative relationship with stroke onset time. Binary logistic regression analysis indicated risk factors of strike on brain-gut axis were NHISS score ≥ 5 and stroke onset time ≤ 24 h. Kruskal–Wallis test results further showed that increasing NIHSS score level meant a higher likely strike on GBA and more possible severe adverse treatment outcome statistically to AIS patients. As a result, the AIS patients with NIHSS score ≥ 5 and stroke onset time ≤ 24 h need to be focused because of the high risk of potential LHI with strike on GBA. Interestingly, AIS patients exhibited much lower energy metabolism than that of healthy controls evidenced by plasma metabolic profiling. In this study, we hypothesize that the first defecating time after admission to the hospital ≥2 days may be considered as a potential risk factor for strike on GBA.

Since both patients and BM pigs with LHI mostly experienced global brain injury and inflammation, we next tried to elucidate the participation of microbiome-gut-brain signaling in the pathophysiology of AIS. Unfortunately, previous experimental findings have limited reference for the clinical diagnosis and treatment of LHI and AIS since the structure and function of a mouse brain differ significantly from humans and large laboratory animals (Zhang et al., 2019). As a research animal with a clear genetic background and an inbreeding coefficient of over 0.8, the Bama miniature pig (BM pig) possesses anatomical and physiological similarities to humans that are greater than those of mice and make the BM pig a favorable substitute for primates in the creation of LHI models (Zhang et al., 2019). Here, BM pig models with LHI were successfully established with ascending pharyngeal artery, a common carotid artery combined with local air injection and confirmed with pathological MRI examination. The symptoms of BM pigs with LHI gradually worsened within 9 h since symptom onset and the new sign of weakness of the left hind limb were derived from gradually expanding necrotic area of cerebral infarction and the surrounding edema area. Left parietal lobe cortex pathological results showed large amounts of edema and necrosis cells around mild dilatational blood vessel, indicating the destruction of the integrity of the NVU, the blood-brain barrier (BBB), and local neuronal necrosis of BM pigs with LHI. Consequently, local and systemic inflammatory responses occurred after the onset of LHI for 9 h, which was verified by markedly upregulated inflammatory cytokines such as *Il-1β* and *Tnf-α*. Consistently, previous studies reported that neuronal apoptosis caused by ischemia stroke releases inflammatory factors and aggravates brain parenchymal damage and BBB destruction.

Increasing evidence has shown that the gut-brain signaling axis is regarded as a key component of stroke outcomes that involve neuroimmune and nervous systems (Rothhammer et al., 2018). In this study, LHI induced marked intestinal epithelial barrier dysfunction and the gut microbiota disruption leading to further damage the central nervous system. Notably, significant downregulation in the ZO-1 and E-cadherin at both protein and mRNAs levels in the ileocecum of BM pigs with LHI suggested that the gut barrier integrity was damaged and permeability was increased due to the intestinal ischemia and reperfusion injury. As a result, release of microbial LPS caused systemic inflammatory response and hematogenous panencephalitis through a pathogen-associated molecular pattern (PAMP) and local DAMP synergy damage (Doll et al., 2015; Hakoupian et al., 2021). Meanwhile, profoundly altered in the gut microbiota composition included upregulation of harmful bacteria and downregulation of beneficial bacteria. Specifically, LHI BM pigs exhibited significant reduction in the relative abundance of two typical probiotics such as *Lactobacillus*, *Bifidobacterium*, and *Butyricicoccus* that are commonly considered as beneficial bacteria capable in producing SCFAs and regulating the immune system (Arboleya et al., 2016; Henrick et al., 2021). Marked upregulation of phylum Proteobacteria and genus *Desulfovibrio* by LHI led to the large amount of LPS releasing into the blood and pass through the damaged BBB. Thus, LPS activated microglia to secrete *Tnf-a*, which could induce neurotoxic phenotype in reactive astrocytes and aggravate the injury of cerebral infarction and ileocecum mucosa barrier.

In addition to intestinal epithelial barrier dysfunction and the gut microbiota disruption by LHI, another prominent outcome of LHI in BM pigs was the profoundly altered microbiota-related metabolites. Here, upregulation in the levels of 5HTP and serotonin in peripheral blood by LHI suggested that LHI led to altered mental status, deliria, rigidity, and myoclonus due to oxidative stress. Previous study showed that high level of blood serotonin reflected oxidative stress and highly contributed to atherosclerosis (Galovic et al., 2021). Owing to the destruction of local BBB, more Trp in the blood of LHI could enter the central nervous system resulting in the increase of Trp in the left cerebral cortex. Damaged local neuronal function caused by LHI affected Trp metabolic steps, resulting in the reduction of serotonin and NAS in brain tissues, in which serotonin is an inhibitory neurotransmitter that could enhance memory and protect neurons from excitatory neurotoxins. NAS is reported as a potent tyrosine kinase receptor B (TrkB) for brain-derived neurotrophic factor (BDNF) ligands and can also inhibit the activation of microglia and remove reactive oxygen species (Galovic et al., 2021). In this study, significant decreases in the levels of both serotonin and NAS in the left parietal lobe cortex further aggravated local brain damage of BM pigs with LHI. Of particular note were the significant changes of many neurotransmitters in plasma and brain tissues of BM pigs with LHI. Marked reduction in the level of glutamine (Gln), a major energy source for intestinal and immune cells, in the plasma of LHI pigs suggested that LHI was an acute severe stress to the whole body affecting the differentiation and regeneration of the digestive tract epithelium. Previous study reported that supplementation of Gln could reduce epithelial cell apoptosis and enhance immune cell function (Yi et al., 2015; Santos et al., 2016). Here, significant downregulation of glutathione (GSH) in the left cerebral cortex, left cerebellum, and white matter indicated that BM pigs with LHI presented an oxidized status with production of reactive oxygen species (ROS) and superoxide (Galovic et al., 2021) that promote the apoptosis of brain cells. Previous studies revealed that acute symptomatic seizures of epilepsy exhibited marked metabolic disturbance of central nervous system, which was further confirmed with marked upregulation of glycine (Gly), serine, dopa, and acetylcholine in brain tissues. In addition, significant upregulation of γ-aminobutyric acid (GABA) in brain tissues of LHI pigs indicated impaired energy metabolism of neurons due to the ischemic injury. Typically, these changed metabolites have been reported to be closely related to gut bacteria. The production of GABA by *Bifidobacterium* and its consequence was accompanied by a decrease in *Actinobacteria*. The levels of SCFA and other neuroactive compounds were consistent with the reduction in the relative abundance of the genus *Bacteroides*.

In conclusion, this study identifies potential LHI patients in the 499 AIS patients with NIHSS score ≥ 5 and stroke onset time ≤ 24 h. More importantly, initial defecation occurring after ≥2 days of hospitalization is considered as one of the key symptoms of GBA dysfunction of stroke. At histopathological level, LHI caused local destruction of the brain with blood occlusion, such as broken BBB and NVU, and consequent global brain inflammation. At the molecular level, LHI stroke on GBA markedly destructed ileocecum mucosa (the gut barrier), resulting in profound disruption in the gut microbial community with downregulations of the abundance of Actinobacteriota and Bacteroidetes and upregulation of Firmicutes, Proteobacteria, and Verrucomicrobia and microbiota-derived metabolites including tryptophan metabolites and neurotransmitters. Strike on GBA in turn ultimately aggravated the systemic inflammatory response and further damaged the central nervous system.

## Data availability statement

The data presented in the study are deposited in National Center for Biotechnology Information GenBank repository (http://www.ncbi.nlm.nih.gov/bioproject/1130385).

## Ethics statement

The studies involving humans were approved by institutional review board of The People’s hospital of Guangxi Zhuang autonomous region (KY-KJT-2019-34). The studies were conducted in accordance with the local legislation and institutional requirements. Written informed consent for participation in this study was provided by the participants’ legal guardians/next of kin.

## Author contributions

DD: Funding acquisition, Writing – original draft, Writing – review & editing. HL: Project administration, Validation, Writing – original draft. ZC: Investigation, Methodology, Writing – original draft. CZ: Investigation, Writing – original draft. RD: Investigation, Writing – original draft. XG: Investigation, Writing – original draft. JW: Investigation, Writing – original draft. YL: Investigation, Writing – original draft. XZ: Investigation, Writing – original draft. LZ: Conceptualization, Funding acquisition, Supervision, Writing – original draft, Writing – review & editing.
