# Supplementary material for: Microbiome-gut-brain axis contributes to patients and Bama miniature pigs with acute large ischemic stroke
Source: Front Neurosci. 2024 Jul 12;18:1378844. doi: 10.3389/fnins.2024.1378844 (PMC11272540; doi:10.3389/fnins.2024.1378844)
Supplement: Supplementary file 1 [file Data_Sheet_1.docx]

**Supplementary materials**

**Microbiome-Gut-Brain Axis Contributes to Patients and Bama Miniature Pigs with Acute Large Ischemic Stroke**

Dazhi Deng ^a,d^, Hehua Lei ^b,c^, Zheng Cao ^b,c^, Cui Zhang ^b,c^, Ruichen Du ^b,c^, Xin Gao ^b,c^, Junjie Wei^e^, Yibo Lu^f^, Xiangzhen Zhou^g^, Limin Zhang ^a,b,c,*^

^a^ Wuhan National Laboratory for Optoelectronics, Huazhong University of Science and Technology, Wuhan 430074, China

^b^ State Key Laboratory of Magnetic Resonance Spectroscopy and Imaging, National Centre for Magnetic Resonance in Wuhan, Innovation Academy for Precision Measurement Science and Technology, CAS, Wuhan 430071, China

^c^ University of Chinese Academy of Sciences, Beijing 100049, China

^d^ Department of Emergency, The People's Hospital of Guangxi Zhuang Autonomous Region & Guangxi Academy of Medical Sciences, Nanning 530021, China

^e^ Department of Neurology, The People's Hospital of Guangxi Zhuang Autonomous Region & Guangxi Academy of Medical Sciences, Nanning 530021, China

^f^ Department of Radiology, Nanning Fourth People's Hospital, Guangxi AIDS Clinical Treatment Center, Nanning 530023, China

^g^ Department of Pathology, The People's Hospital of Guangxi Zhuang Autonomous Region & Guangxi Academy of Medical Sciences, Nanning 530021, China

* Correspondent author: zhanglm@wipm.ac.cn (L. Zhang)

**Materials and methods**

*^1^H NMR-based serum metabolic profiling of AIS patients and healthy controls*

Serum samples of AIS patients and healthy controls were prepared by mixing 200 μL serum with 400 μL saline solution containing 10% D2O. Following vortexing and centrifugation (11180g, 10 min and 4 °C), 550 μL samples was transferred into 5 mm NMR tubes. A water-presaturated Carr-Purcell-Meiboom-Gill (CPMG) pulse sequence (recycle delay-90°-(τ-180°-τ)_n_-acquisition) was employed to attenuate NMR signals from macromolecules. 90° pulse length was adjusted to about 10 μs for each sample and water signal was suppressed with a weak continuous wave irradiation during recycle delay (RD). Data points (64 K) were collected for each spectrum with a spectral width of 20 ppm (12 kHz) and RD of 2 s. The spin−spin relaxation delay, 2nτ, was set to 96 ms. Free induction decays so obtained for all samples were multiplied by an exponential function with a line broadening factor of 1 Hz prior to Fourier transformation. Chemical shifts for all spectra were referenced to the anomeric proton signal of α-glucose (δ5.23). For the purposes of signal assignments, a series of two-dimensional NMR (2D NMR) spectra were recorded and processed for selected samples. These spectra included ^1^H−^1^H correlation spectroscopy (COSY), total correlation spectroscopy (TOCSY), ^1^H−^13^C heteronuclear single quantum correlation (HSQC) and ^1^H−^13^C heteronuclear multiple bond correlation (HMBC) spectra.

All the ^1^H NMR spectra were corrected for phase and baseline distortions using Topspin (V2.0, Bruker Biospin) and the spectral region δ 0.5−9.5 were divided into buckets with equal width of 0.004 ppm (2.4 Hz) using AMIX software package (V3.8.3, Bruker Biospin). The regions at δ 4.32−5.5 was discarded to eliminate the effects of imperfect water saturation. Multivariate data analysis was conducted with SIMCA-P+ package (V14.0, Umetrics, Sweden) following normalization to the volume of serum samples. Principal component analysis (PCA) was carried out on the mean-centered data to generate an overview and check for the outliers. Partial least-squares discriminant analysis (PLS-DA) and the orthogonal projection to latent structure with discriminant analysis (OPLS-DA) were subsequently performed using the unit-variance scaled data to find metabolites having significant intergroup differences. The OPLS-DA models were built with two components calculated and with 6-fold cross-validation. These models were further evaluated for their validities with CV-ANOVA method. After back-transformation, the loadings were plotted using an inhouse developed Matlab (V7.8, The Mathworks, MA) script with correlation coefficients color-coded for each variable (or the metabolite signals). The color-coded variables indicate the significance of metabolites contributing to the intergroup differentiation with a “hot” colored (e.g., red) metabolite being more significant than a “cold” colored (e.g., blue) ones. Cutoff values for the correlation coefficients were chosen depending on the number of samples used to extract metabolites having significant intergroup differences based on the discrimination significance (p < 0.05) for the Pearson’s product-moment correlation coefficients.

*Quantitative analysis of plasma and brain tryptophan metabolites*

Tryptophan metabolites extraction were performed from plasma and brain tissues of Bama Miniature Pigs. Samples mixed with internal standard (10 μL d_5_-TRP) were respectively homogenized with 400 μL cooled methanol and 50 μL acetonitrile: water solution (1:1 v/v) containing 0.1% formic acid using the Qiagen Tissue-Lyser (Retsch GmBH, Germany) at 20 Hz for 90 s. After extraction for two times, the combined supernatants were collected and evaporated into dryness following centrifugation. Serum sample (10 μL) was uniformly mixed with 10 μL of internal standard (d_5_-trp), 150 μL cooled methanol and 10 μL acetonitrile: water solution (1:1 v/v) containing 0.1% formic acid. After centrifugation for 20 min (4 °C), the supernatants were collected and lyophilized for removing methanol in vacuum. Dried extracts were reconstituted in 100 μL of acetonitrile: water solution (1:1 v/v) containing 0.1% formic acid. Qualitative and quantitative analyses of tryptophan metabolites were performed using an ultrahigh performance liquid chromatography (Agilent 1290) coupled with a 6460 triple quadrupole mass spectrometry (UHPLC-QQQ-MS, Agilent Technologies, Inc.). The precursor ions of tryptophan metabolites were pre-scanned through multiple reaction monitoring (MRM) of all sample mixtures and the structures were identified through MS/MS spectra. Quantification of tryptophan metabolites was performed using calibration curves based on MRM and the ratios of the integrated peak areas of tryptophan metabolites and internal standards.

Table S1. The primer sequences of qPCR used in this study.

| Name | Forward primer (5’ -3’) | Reverse primer (5’-3’) |
| --- | --- | --- |
| IL-1β | GGCCATAGTACCTGAACCCG | TTGGGTGCAGCACTTCATCT |
| TNF-α | GGCCCAAGGACTCAGATCAT | GGCATACCCACTCTGCCATT |
| E-cadherin | CGACGGTGTGGTTACAGTCA | AGAGGGAGAGTCCTGATGGC |
| ZO-1 | GATGTTTATGCGGACGGTGG | CATTGCTGTGCTCTTAGCGG |
| β-actin | TCAGCAAGCAGGAGTACGAC | GGAATGCAACTAACAGTCCGC |
| GAPDH | GTCGGAGTGAACGGATTTGGC | GGAGGTCAATGAAGGGGTCA |

Table S2. NMR assignments for the metabolites in human serum.

| Key | metabolites | Moieties | δ ^1^H (ppm) and multiplicity ^a^ | δ ^13^C (ppm) |
| --- | --- | --- | --- | --- |
| 1 | HDL | CH_3_ | 0.82(m) | b |
| 2 | LDL | CH_3_ | 0.85(m) | b |
| 3 | VLDL | CH_3_ | 0.88(m) | b |
| 4 | Leucine | αCH  βCH_2_  γCH  δCH_3_  δ′CH_3_ | 3.74(t)  1.69(m)  1.72(m)  0.97(d)  0.96(d) | 56.0  27.2  42.8  24.4  24.4 |
| 5 | Isoleucine | αCH  βCH  γCH_2_  γ′CH_3_  δCH_3_ | 3.67(d)  1.98(m)  1.27(m),1.47(m)  1.01(d)  0.94 (t) | 62.11  38.4  27.7  17.4  14.2 |
| 6 | Valine | αCH  βCH  γCH_3_ | 3.62(d)  2.28(m)  1.05(d) | 63.2  31.9  20.8 |
| 7 | D-3-hydroxybutyrate | CH  CH_2_  γCH_3_  CH_2_ | 4.16(dt)  2.41(dd)  1.20(d)  2.31(dd) | 68.8  49.5  24.4  49.5 |
| 8 | Lactate | αCH  βCH_3_  COOH | 4.12(q)  1.33(d) | 71.2  22.4  184.9 |
| 9 | Alanine | αCH  βCH_3_  COOH | 3.78(q)  1.48(d) | 53.5  18.9  178.8 |
| 10 | Lysine | αCH  εCH_2_  βCH  γCH_2_  δCH_2_  COOH | 3.76(t)  3.03(t)  1.92(m)  1.72(m)  1.45(m) | 64.1  42.2  33.4  29.9  23.5  177.0 |
| 11 | Arginine | γCH_2_  βCH_2_  δCH_2_  αCH | 1.69(m)  1.92(m)  3.25(t)  3.76(t) | b |
| 12 | Acetate | CH_3_ | 1.91(s) | 26.5/184.4 |
| 13 | N-acetyl-glycoproteins  (NAG) | CH_3_ | 2.04(s) | b |
| 14 | Glutamate | αCH  βCH_2_  γCH_2_  COOH  COOH | 3.76(m)  2.06(m)  2.12(m)  2.35(m) | 57.4  29.8  36.2  177.4  184.0 |
| 15 | Glutamine | αCH  βCH_2_  γCH_2_  COOH  CO | 3.77(t)  2.14(m)  2.46(m) | 57.2  29.0  34.0  b  180.3 |
| 16 | Citrate | CH_2_  CH_2_  C-OH  COOH  COOH | 2.55(d)  2.69(d) | 48.1  47.9  78.2  181.7  184.4 |
| 17 | Unsaturated fatty acids | CH | 6.53(s) | 137.8 |
| 18 | Lipid | CH_3_  (CH_2_)_n_  CH_2_-CH=CH  CH_2_-CO  =C-CH_2_-C=  CH=CH | 0.89(m)  1.27(m)  2.0(m)  2.3(m),  2.78(m)  5.3(m) | b |
| 19 | Choline | N(CH_3_)_3_  NCH_2_  OCH_2_ | 3.21(s)  3.53(m)  4.07(m) | 56.7  70.3  58.2 |
| 20 | Phosphoryl-choline  (PC) | N(CH_3_)_3_  NCH_2_  OCH_2_ | 3.22(s)  3.60(m)  4.17(m) | 56.4  69.3  60.8 |
| 21 | Glycerophospho-choline  (GPC) | N(CH_3_)_3_  NCH_2_  OCH_2_ | 3.24(s)  3.69(m)  4.33(m) | 56.4  b  b |
| 22 | Glucose/amino acids | α-CH resonances | 3.3-3.9 | b |
| 23 | Scyllitol | CH | 3.36 | b |
| 24 | Glycine | CH_2_  COOH | 3.56(s) | 44.3  175.3 |
| 25 | β-glucose | 1-CH | 4.66(d) | 98.6 |
| 26 | α-glucose | 1-CH | 5.23(d) | 94.8 |
| 27 | Tyrosine | βCH_2_  αCH  3 or 5-CH  2 or 6-CH | 3.06,3.14(dd)  3.94(t)  6.90 (d)  7.20(d) | 38.3  57.4  119.1  134.0 |
| 28 | Histidine | CH  CH | 7.09(s)  7.89(s) | 120.2  139.1 |
| 29 | Phenylalanine | CH_2_  CH_2_′  N-CH  2 or 6-CH  3 or 5-CH  4-CH | 3.14(dd)  3.28(dd)  3.99(dd)  7.33(m)  7.40(m)  7.38 (m) | b  b  b  132.4  132.2  131.1 |
| 30 | Formate | CH | 8.46(s) | 174.3 |
| 31 | α-mannose | 1. CH 2. CH | 5.18(d)  3.93(m) | 97.1  75.3 |
| 32 | Acetylcarnitine | CH3C=O  α-CH  α-CH′  γ-CH2 | 2.13(s)  2.46(m)  2.63(m)  3.90 (m) | b |
| 33 | Myo-inositol | 1,3-CH 2-CH 4,6-CH | 3.65(m)  3.29(m)  3.57(m) | b |
| 34 | Triglycerides | CHO  CH2O  CH2'O | 5.21(m)  4.07(m)  4.28(m) | 69.9  62.8  62.8 |

^a^ Key: s, singlet; d, doublet; t, triplet; q, quartet; m, multiplet; dd, doublet of doublet.

^b^ The signals or the multiplicities were not determined.
